# How to finance national antimicrobial resistance action plans

**DOI:** 10.2471/BLT.24.291638

**Published:** 2024-03-27

**Authors:** Serife Genc Ileri, Ritu Sadana, Anand Balachandran

**Affiliations:** a Department of Economics, Faculty of Management, Istanbul Technical University, Macka Campus, 34367 Macka Istanbul, Türkiye.; b Secretariat, WHO Council on the Economics of Health for All, World Health Organization, Geneva, Switzerland.; c Surveillance, Prevention and Control Department, World Health Organization, Geneva, Switzerland.

According to the World Health Organization (WHO), antimicrobial resistance, which occurs when bacteria, viruses, fungi and parasites change and stop responding to medication, is one of the top 10 health threats facing the world.^1^ Estimates from 2019 suggest that 1.27 million deaths are attributable to antimicrobial resistance (more than human immunodeficiency virus/acquired immunodeficiency disease or malaria deaths), with 10 million deaths per year estimated by 2050.^2^ Without action, antimicrobial resistance will push an additional 28.3 million people into poverty – due to its disproportionately negative impact on various outcomes such as economic output, health-care costs and fiscal burden in low-income countries – and will lead to a decrease of 3.8% in global gross domestic product (GDP) by 2050.^3^ Comparing such projections to the 3.1% decline in global GDP in 2020,^4^ at the height of the coronavirus disease 2019 (COVID-19) restrictions, underscores the need for additional investments to address antimicrobial resistance.

Despite this high cost of inaction, several obstacles have prevented policy-makers from focusing on this threat. These obstacles include the complexity of addressing antimicrobial resistance across sectors, weak political incentives, and inadequate data and analysis, such as a lack of cost–benefit analyses relevant to different sectors. The lack of trust and transparency in governance systems also result in limited financing of national antimicrobial resistance budgets. This article focuses on more and better finance advocated by the WHO Council on the Economics of Health for All,^5^ details why a whole-of-government approach is necessary to finance antimicrobial resistance and outlines what steps can be taken.

Antimicrobial resistance is a One Health challenge involving multiple sectors, and requires a whole-of-government approach and national plans to align human, animal, agriculture and planetary health objectives. Each sector, however, has unique perspectives, and may have conflicting goals in relation to addressing antimicrobial resistance. Moreover, health promotion initiatives between different tiers and departments of government, including antimicrobial resistance, could create unequal incentives and rewards.^6^ Agriculture, food–animal and trade sectors contribute to the transmission of resistant organisms, within and across countries. Antimicrobials are used in livestock husbandry, both for treatment purposes and to promote growth, with global procurement estimated to be 73%–100% higher than purchases for human health.^7^ Excessive use of antibiotics also reflects increasing global demand for meat. Moreover, overuse in humans is due to a misunderstanding that antibiotics help with viral diseases such as the common cold, influenza or COVID-19, which is false.

At the global level, trade policies could mitigate antimicrobial resistance burden through bans, user fees and restrictions on antimicrobials used in raising food animals.^8^ Yet at the national level, competing interests between ministries are apparent. This context underlines the challenge of addressing antimicrobial resistance if it fails to engage all related government entities that have a direct role in mitigation. Although the benefits of investments and the costs of inaction in response have been documented for the human health sector, they are not necessarily documented for other sectors. To address this gap, the quadripartite organizations (Food and Agriculture Organization, World Organisation for Animal Health, WHO and the United Nations Environment Programme) have undertaken work to estimate the global costs of antimicrobial resistance and the associated benefits of response across different sectors based on a core package of interventions.

At the national level, countries have been taking steps towards making goals and actions related to antimicrobial resistance explicit, with an increasing number of countries developing national action plans to address antimicrobial resistance. Latest data from the 2023 Tracking Antimicrobial Resistance Country Self-assessment Survey reveal that from 177 countries surveyed, 163 developed a plan. But having a plan does not guarantee implementation, as only about 11% (20/177) countries with a national action plan have made financial provisions in their national budgets to implement their respective plan.^9^ This low figure reflects limited domestic resources and dependency on project-based aid. 

## Approaches to increase finance

Providing financial resources to national action plans on antimicrobial resistance requires politicians and other policy-makers to focus on achieving what matters to people and the planet over the long term.^10^

As a first step, countries should invest in establishing national antimicrobial resistance governance and multisectoral coordination mechanisms, to set priorities for actions, cost national action plans, allocate resources and monitor progress. This multisectoral mechanism can direct, mainstream and finance national action plans through existing national sectoral plans and budgets towards sustainability. For example, Denmark founded a National Antibiotic Council with stakeholders in 2010 to coordinate action and support the health ministry in setting priorities, and earmarked pooled funds for promising antimicrobial resistance projects in the period 2014–2016.^11^ In the human health sector, financing antimicrobial resistance interventions can also fit into existing health sector plans and budgets such as those for health-emergency preparedness, response and resilience, health workforce and overall health system strengthening efforts.

As a second step, concrete mechanisms are needed to incorporate predictable financial resources for national action plans into budgets and planning cycles of national governments and national and regional development banks; and antimicrobial resistance interventions into global financing mechanisms such as the Global Fund, the Pandemic Fund and Global Financing Facility for the Environment. Doing so should enable a broad approach to increase fiscal space and finance rather than relying on siloed or vertical approaches. Ministries and agencies that coordinate and collaborate closely with the finance ministry can reduce inefficiencies, increase predictable financing and improve outcomes. This approach aligns investments and expenditures on agreed high priorities and national goals, and informs budgeting processes of ministries and agencies to achieve results. For example, the Slovak government spotted potential for improvement and asked finance and health ministries to conduct a health-care spending review, resulting in some 730 million euros detected between 2020 and 2021 where savings could be made and allocated to national priorities, including action on antimicrobial resistance.^12^

Moreover, the *WHO implementation handbook for national action plans on antimicrobial resistance: guidance for the human health sector*^13^ and the WHO costing and budgeting tool offer guidance on processes for prioritization and budgeting. Other intersectoral challenges show that tools, such as outcome-based budgeting, earmarked and delegated financing, pooled and aligned budgeting, are also useful in financing and budgeting multisectoral issues. For example, the 2017–2021 WHO Country Cooperation Strategy of Thailand pooled budgets from five national partners and WHO, and channelled these towards addressing antimicrobial resistance. Similarly, Singapore’s One Health Coordinating Committee, comprised of different national agencies, created and funded jointly its One Health antimicrobial resistance research. These tools and mechanisms also help in showing the relevant trade-offs and funding decisions, and could support coordinating and financing budgets around the challenge of antimicrobial resistance.

These and other country examples will be further discussed in a forthcoming publication from the WHO Council on the Economics of Health for All, to be released before the September 2024 United Nations General Assembly High-Level meeting on antimicrobial resistance. This meeting should also provide a forum to advance the call for sustainable financing by WHO Member States, expressed in resolutions addressing antimicrobial resistance adopted at the World Health Assembly in 2019,^14^ as well as in reports by the Director-General to Member States in the 76th World Health Assembly and in the recent 154th Executive Board; many of these ideas are also echoed by the Global Leaders Group on antimicrobial resistance. Another resolution is expected to be adopted in 2024.

Antimicrobial resistance merits policy that draws on financial and economic levers that recognize governments must discard narrow-sighted practices, invest in their capacity to set a direction for development, and ensure accountability in the short and long-term health of people and the planet. Antimicrobials are a pillar of modern medicine; addressing antimicrobial resistance is key to a whole-of-government approach that cannot be allowed to fail.
